# Bronchial Schwannoma Incidentally Discovered via Bronchoscopy: A Case Report

**DOI:** 10.7759/cureus.38011

**Published:** 2023-04-23

**Authors:** Kyle A Burton, Matthew Karulf, Brian Pahn

**Affiliations:** 1 Internal Medicine, Michigan State University College of Human Medicine, Marquette, USA; 2 Pulmonology, Upper Peninsula Health System, Marquette, USA; 3 Pathology, Upper Peninsula Health System, Marquette, USA

**Keywords:** spindle cells, endobronchial lesion, bronchoscopy, lung tumor, bronchial schwannoma

## Abstract

Bronchial schwannomas are rare tumors that arise from Schwann cells and account for a very small percentage of primary lung tumors. This case report describes a rare incidental finding of a bronchial schwannoma discovered in the left lower lobe secondary carina via bronchoscopy in a 71-year-old female who presented with minimal symptoms.

## Introduction

Schwannomas are benign tumors arising from the Schwann cells of the peripheral nervous system. Schwannomas are most commonly found in the upper limbs, the head, the trunk, and the flexor surfaces of the lower extremities [1]. Schwannomas presenting in the bronchial tree are an extremely rare finding. When symptomatic, patients with tracheal or bronchial schwannomas may present with coughing, wheezing, and dyspnea [2]. We describe an asymptomatic patient with chronic obstructive pulmonary disease (COPD) who was referred to pulmonology for evaluation of an abnormal CT chest. She subsequently underwent bronchoscopy which revealed a polyp that was not appreciated on imaging. Further histologic examination confirmed the diagnosis of a bronchial schwannoma.

## Case presentation

**Figure 1:**
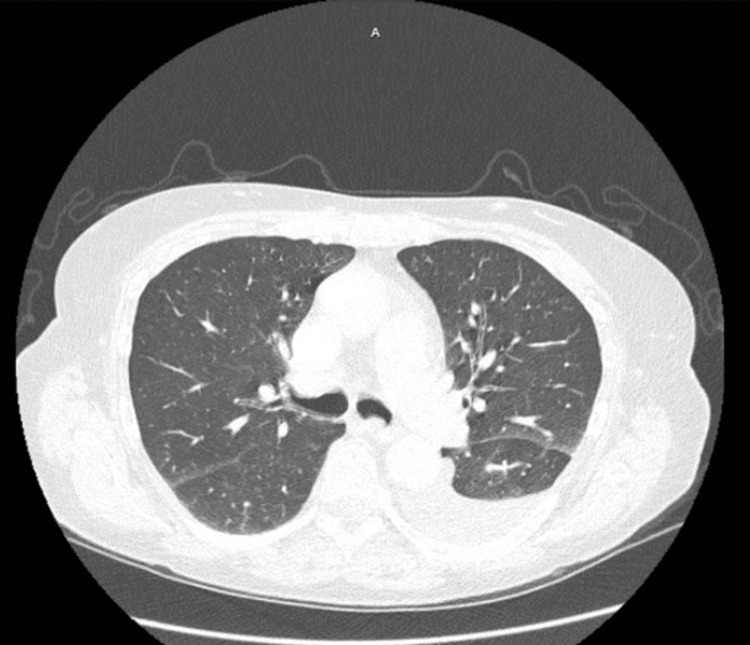
CT chest identifying left mainstem endobronchial debris which is likely a mucoid secretion

**Figure 2:**
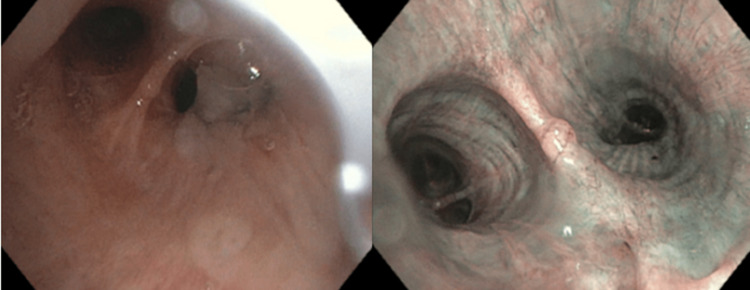
A 2mm polyp identified via bronchoscopy at the left lower lobe secondary carina

A 71-year-old Caucasian female with a past medical history of COPD, hyperlipidemia, peripheral vascular disease, and syndrome of inappropriate antidiuretic hormone was referred to pulmonology for evaluation of an abnormal CT chest that had identified left mainstem endobronchial debris versus tumor. At the time of pulmonology evaluation, the patient was mostly asymptomatic, presenting with only a chronic smoker’s cough with occasional phlegm production. Given that this patient had a history of one pack of cigarettes every day for 50 years, a repeat CT chest was obtained which showed a persistent left mainstem endobronchial lesion as shown in Figure 1, prompting further evaluation via bronchoscopy. She subsequently underwent bronchoscopy which revealed mucoid secretions in multiple bilateral subsegmental bronchi including the right middle lobe, right lower lobe, left lower lobe, and lingula. A mucoid secretion was likely the appreciated CT chest finding that precipitated a referral to pulmonology for evaluation. A detailed examination of the airway was completed after clearance of the airway, which incidentally revealed a 2 mm pink polyp located at the left lower lobe secondary carina as shown in Figure 2. This polyp was too small to be appreciated on the CT chest. Three endobronchial biopsies of this 2 mm polyp were obtained.

**Figure 3:**
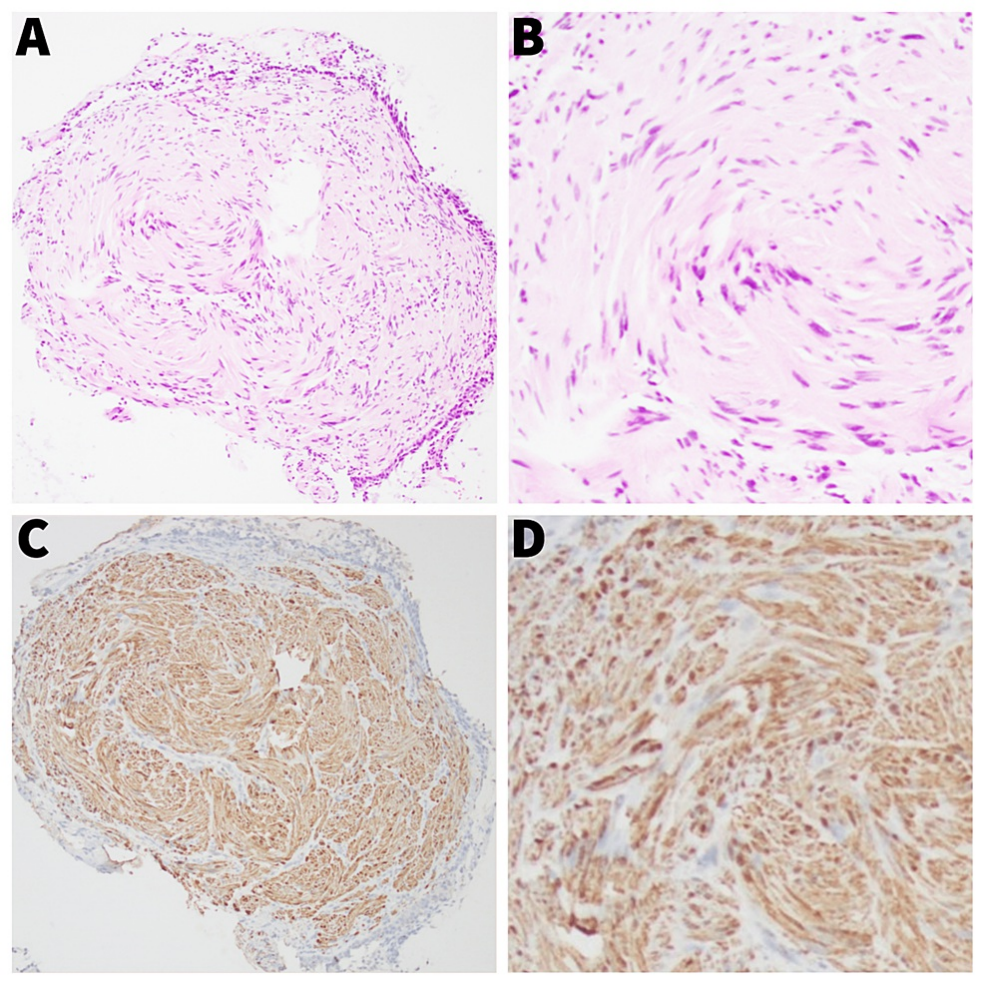
A, B: Hematoxylin and eosin staining; C, D: S100 immunohistochemical staining

Histologic examination revealed well-defined submucosal, bland spindle cell proliferation with cells arranged in vague fascicles and demonstrating tapered nuclei with focal palisading and eosinophilic cytoplasm as shown in Figure 3. Immunohistochemical staining revealed S100 positivity. Given the palisading spindle cell morphology and S100 positivity, this tumor was characterized as a schwannoma.

## Discussion

Schwannomas have been documented to occur throughout the body, including the head, neck, trunk, and extremities [1]. The tumors are typically benign and can be asymptomatic, which may make diagnosis challenging. Schwannomas presenting in the tracheobronchial tree are a rare presentation of these tumors, occurring only in approximately 0.2% of primary tumors of the trachea and bronchus [3].

Schwannomas presenting in the bronchial tree are typically diagnosed in adults and may remain asymptomatic until they have grown to a significant size [4]. In some cases, they can enlarge enough to cause a complete bronchial obstruction, as documented in at least one case report [5].

Histopathological examination is essential for the definitive diagnosis of bronchial schwannomas. Microscopically, these tumors are characterized by spindle cells arranged in fascicles, with nuclear palisading. Additionally, positive staining for S-100 protein helps to support the diagnosis [6].

Bronchial schwannomas are best treated with surgical resection, which offers a favorable prognosis and low risk of recurrence [7]. In cases where surgical resection is not feasible, radiation therapy has been reported in the literature as a successful alternative treatment option [8].

## Conclusions

Bronchial schwannoma should be considered as a possible differential diagnosis of pulmonary masses, even in asymptomatic patients. Small bronchial schwannomas may not be detected through non-invasive imaging and patients may not exhibit symptoms until the tumor has grown to a significant size. The prognosis for benign tumors is excellent, with complete surgical resection as the preferred treatment option.
